# Ex Vivo Confocal Laser Scanning Microscopy for Real‐Time Pattern and Single‐Cell Analysis in Inflammatory Skin Diseases

**DOI:** 10.1111/exd.70179

**Published:** 2025-11-17

**Authors:** D. Hartmann, L. Stärr, M. Maurer, Y. Stohldreier, L. Buttgereit, A. Swarlik, E. C. Sattler, L. E. French, M. Deußing

**Affiliations:** ^1^ Department of Dermatology and Allergy LMU University Hospital, LMU Munich Munich Germany; ^2^ Department of Dermatology and Allergy Munich Municipal Hospital Munich Germany; ^3^ Department of Neuroradiology LMU University Hospital, LMU Munich Munich Germany; ^4^ Department of Dermatology and Cutaneous Surgery, Miller School of Medicine University of Miami Miami Florida USA

## Abstract

Inflammatory skin diseases are common and often difficult to differentiate. Ex vivo confocal laser scanning microscopy (EVCM) offers a rapid and promising approach. This study aimed to assess the diagnostic utility of EVCM in differentiating inflammatory dermatoses, particularly eczema, psoriasis and lichen planus, by comparing its performance with gold standard histopathology. Tissue samples of 110 patients presenting with inflammatory skin conditions were subjected to both EVCM and conventional histopathology. EVCM images were analysed by three blinded observers, with varying knowledge in histopathology and EVCM, utilising pattern analysis based on Ackermann's classification and single‐cell analysis focusing on neutrophil (neutrophils) and eosinophil (eosinophils) granulocytes. Sensitivity and specificity were calculated using contingency tables. We used Cohen's Kappa coefficient and Firth's logistic regression models to evaluate the correlations between disease‐associated histopathological features observed via EVCM and histopathology, as well as their impact on accurate histopathological diagnoses. Our findings demonstrate that EVCM provides rapid and insightful visualisation of characteristic features associated with inflammatory dermatological diseases. Diagnostic accuracy varied based on observer experience. The specialist proficient in both EVCM and histopathology achieved the highest accuracy rates for correctly diagnosing lichen planus (97.27%), psoriasis (95.45%) and eczema (92.73%). In conclusion EVCM emerges as a promising adjunct to histopathology, offering a swift and meaningful visualisation of inflammatory disease features. The integration of EVCM could significantly contribute to expediting diagnostic workflows and facilitating prompt, targeted therapeutic interventions. Further research and validation are warranted to establish EVCM's role in routine clinical practice.

## 1 Introduction

Inflammatory dermatological diseases, encompassing conditions such as eczema, psoriasis and lichen planus, pose a diagnostic challenge due to their often similar and overlapping clinical presentations [1]. Timely and accurate diagnosis is crucial for initiating appropriate therapeutic interventions and improving patient outcomes. While histopathology has long been the gold standard of histopathological assessment, advancements in imaging technologies offer new avenues for enhancing diagnostic precision. Additionally, these innovations facilitate faster diagnoses, offering a substantial benefit in both diagnostic and everyday clinical practice [2].

Ex vivo confocal laser scanning microscopy (EVCM) has emerged as a promising imaging modality, offering nearly real‐time, high‐resolution visualisation of skin structures at the cellular level [3]. Previous studies have highlighted the diverse utility of EVCM, initially implemented for rapid intraoperative margin control in non‐melanoma skin cancer, especially basal cell carcinoma [4, 5, 6]. Over time, this technique has been applied to the evaluation of a wide range of skin alterations, including melanocytic lesions [7, 8], squamous cell carcinomas [9] and other inflammatory dermatoses [10, 11, 12].

As stated by A.B. Ackermann inflammatory infiltrates can be classified according to their architectural and cytological criteria. Ackermann distinguishes between patterns that arise either exclusively from inflammatory cells or in combination with changes in the epidermal and adnexal epithelium. A distinction is also made according to the category of inflammatory cell types. This differentiation makes it possible to identify eight essential inflammatory patterns, which are still of great importance in histopathology [13, 14].

The utilisation of pattern analysis based on Ackermann's classification and a focused single‐cell analysis adds a layer of specificity to the evaluation of EVCM images. This comparative analysis aims to elucidate the diagnostic capabilities of EVCM in discerning distinct patterns associated with eczema, psoriasis and lichen planus, potentially paving the way for its integration into routine clinical practice. By comparing EVCM with the established gold standard, our objective was to evaluate the efficacy of this novel technique in providing rapid and meaningful insights into the characteristic features of these conditions.

The findings from this research were to further explore and validate EVCM's role in expediting the diagnosis of inflammatory dermatological diseases.

## 2 Materials and Methods

### 2.1 Study Participants

Participants were recruited at the Department of Dermatology and Allergy of the University Hospital at Ludwig‐Maximilians‐University (LMU) in Munich between March 2023 and April 2024. Patients included had skin types Fitzpatrick type I to IV. Before inclusion in the study, each patient gave written informed consent. The study was approved by the local ethics committee of the LMU university hospital (Ref.‐Nr. 19‐150 and Ref.‐Nr. 23‐0393).

Patients presenting with inflammatory dermatological diseases, clinically suspected as atopic eczema, psoriasis or lichen planus underwent standard biopsy procedures (4 mm punch biopsy), resulting in 110 tissue samples. Localization of the lesions were on the patients' head (*n* = 9), thorax (*n* = 37), arms (*n* = 32) and legs (*n* = 32).

### 2.2 Ex Vivo Confocal Laser Scanning Microscopy (EVCM)

Prior to imaging, all skin samples underwent a standardised staining protocol including acridine orange (0.1 mmol/L, Sigma‐Aldrich, St. Louis/MO, USA) followed by phosphate‐buffered saline (0.1 mmol/L, Dulbecco's Phosphate Buffered Saline; PBS; pH 7,4) and citric acid (0.1 mmol/L) for a so called aceto‐whitening for 30 s each [15]. All samples were fully immersed in the staining solutions, covering the entire surface of the cutting edge.

EVCM imaging was performed using the Vivascope 2500 G‐4 device (Vivascope, Munich, Germany) with two laser wavelengths at 488 nm (blue) and 638 nm (red). Therefore, the tissue probes were placed on object slides (R. Langenbrinck, Emmendingen, Germany), mounted with sponges as well as magnets, and sectioned in vertical mode to reveal all skin layers according to standard histopathological procedures [16, 17].

### 2.3 Histological Analysis and Correlation

Notably, the sample remained unaltered and intact during the EVCM imaging process, allowing it to be preserved and subsequently prepared for routine histopathological investigation [2]. After imaging, the skin samples were immediately fixed in 4% buffered formalin and subsequently processed for routine paraffin embedding. Haematoxylin and eosin staining was employed for morphological evaluation [18].

Histopathological assessment was performed by experienced histopathologists not aware of the EVCM imaging data.

### 2.4 EVCM Image Analysis

Overview and detailed digital HE (DHE) EVCM images [19, 20] were presented in a randomised order to three blinded observers, an EVCM‐trained histopathologist = observer 1 (D.H.), an EVCM‐trained dermatologist with no experience in histopathology = observer 2 (M.D.) and an EVCM‐unexperienced dermatologist in histopathological training = observer 3 (M.M.).

The following morphologic EVCM features were analysed:

1. Stratum corneum: hyperkeratosis, parakeratosis and orthokeratosis.
2. Epidermis: acanthosis, spongiosis, sawtooth‐like hyperplasia, psoriasiform hyperplasia, non‐specific hyperplasia or normal epidermis.
3. Inflammatory patterns: perivascular, diffuse, band‐like infiltrate or absence of infiltrate.
4. Single‐cell analysis: predominance of either neutrophils, eosinophils or lymphocytes.

Subsequently, the investigators were instructed to categorise the images into following disease categories: eczema, psoriasis or lichen planus. Results obtained from EVCM images were then compared to traditional histopathology to determine the concordance and discrepancy between the two diagnostic modalities.

Statistical analyses were conducted using R version 4.4.1 (R Foundation for Statistical Computing, Vienna, Austria) with RStudio Build 764. All tests were two‐sided, with a significance level of α = 0.05. The Shapiro–Wilk test was used to assess normality of the data. For metric variables that were normally distributed, results were presented as mean ± standard deviation, whereas those not following a normal distribution were reported as median (interquartile range). To evaluate associations between categorical variables, Fisher's exact test was used when the sample size in any group was less than 5; otherwise, the *χ* ^2^ test was used. Comparison between the three disease groups were done with the Kruskal‐Wallis rank sum test.

We conducted an analysis using Cohen's kappa statistic, to evaluate the concordance between features observed in EVCM images and gold standard histopathology. Three observers assessed EVCM images of the inflammatory skin diseases focusing on specific histopathological categories: stratum corneum, epidermis, inflammatory infiltrate and single cells. Those observations were then compared to the corresponding findings in histopathology.

Agreement levels were interpreted based on established thresholds: *k* = 0–0.20 (slight agreement), 0.21–0.40 (fair agreement), 0.41–0.60 (moderate agreement), 0.61–0.80 (substantial agreement) and 0.81–1.00 (almost perfect agreement) [21]. Initially, overall agreement for each main category was assessed by pooling all associated subcategories. Subsequently, individual features within each main category were analysed separately to identify specific correlations.

To analyse the relationship between disease‐associated histopathological features detected in EVCM images and diagnostic outcomes, we applied Firth's multivariable regression models, also addressing separation issues [22].

We examined the association between features identified by the three observers in EVCM images and their ability to make the correct diagnosis. The dependent variable was the observers' correct diagnosis, while the independent variables included observed features from key histopathological categories in the stratum corneum, epidermis, inflammatory infiltrate and single cells.

The most frequently observed feature within each category was designated as the reference category (parakeratosis as reference category for the stratum corneum, psoriasiform hyperplasia for the epidermis, perivascular infiltrate for inflammatory infiltrate and lymphocytes for single‐cell types). The reported coefficients (coeff) represent the log odds ratios, providing insights into how specific features influenced diagnostic accuracy.

## 3 Results

### 3.1 Desc. Statistics Cohort

The study included 110 patients (*n* = 58 female, mean age 53.84 (36.95–63.92) years) with inflammatory dermatological diseases (eczema 56.36% (62/110), psoriasis 25.45% (28/110), lichen planus 18.18% (20/110)). No statistically significant differences were observed when comparing age, sex or biopsy location across the three diagnoses (*p* > 0.05). Descriptive statistics for the patient population are summarized in Table S1.

### 3.2 Contingency Tables

When comparing the diagnoses made using EVCM to those established by gold standard histopathology, observer 1 correctly diagnosed 92.72% (102/110) of all cases, representing the highest number of accurate diagnoses. Observer 2 correctly identified 70.19% (78/110), while observer 3 made 61.82% (68/110) correct diagnoses.

**FIGURE 1:**
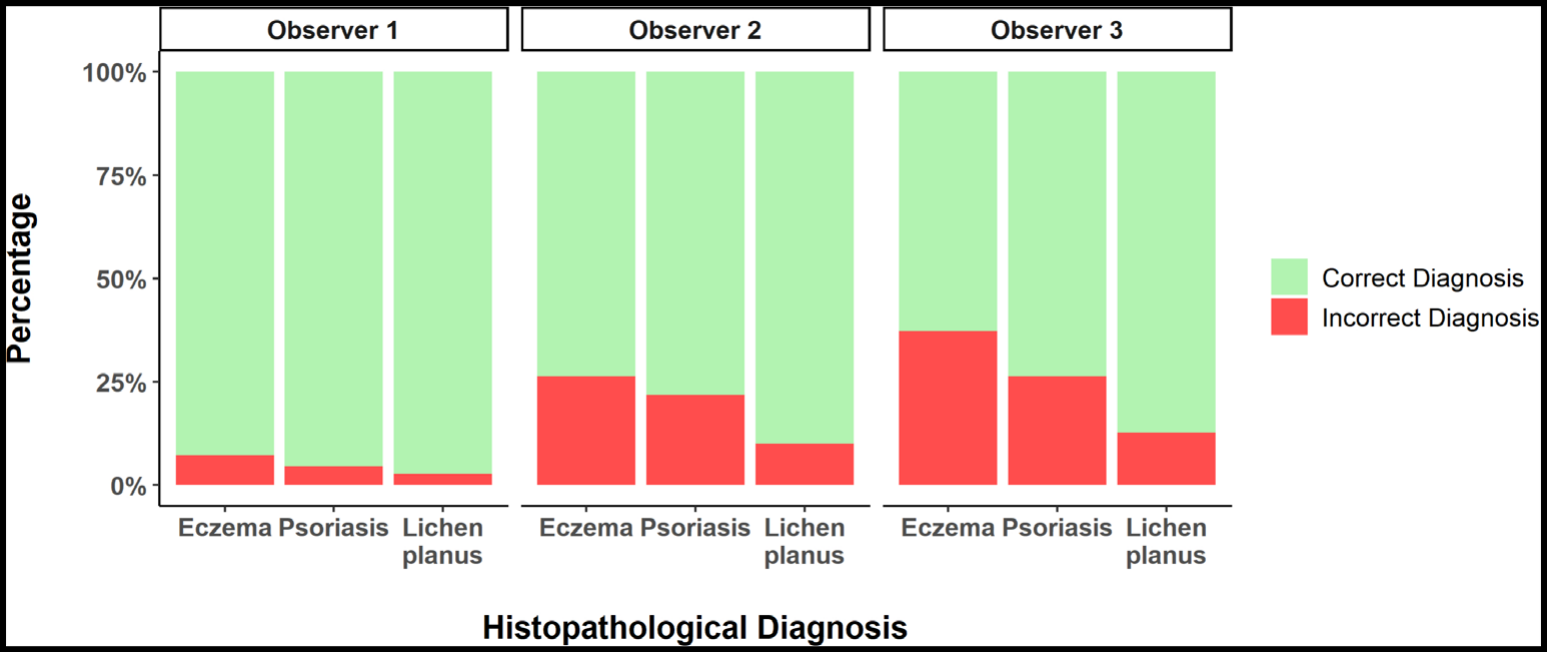
This histogram compares the accuracy of diagnoses by all three observers based on ex vivo confocal laser scanning microscopy (EVCM) images against histopathological diagnoses for eczema, psoriasis and lichen planus. Green bars show correct diagnoses, while red bars indicate incorrect ones.

To evaluate diagnostic performance across all three skin diseases, we analysed the accuracy, sensitivity and specificity for all three observers. Observer 1 consistently achieved the highest accuracy across all conditions (lichen planus: 97.27%, psoriasis: 95.45%, eczema: 92.73%), followed by observer 2 (90.00%, 78.18%, 73.64%) and observer 3 (87.27%, 73.64%, 62.73%, Figure 1). A detailed summary of these metrics is provided in Table 1.

**TABLE 1:** Diagnostic performance metrics for the three observers across the three skin diseases.

Disease	Observer	Accuracy (%)	Sensitivity (%)	Specificity (%)	PPV (%)/NPV (%)
Lichen planus	Observer 1	97.27	85.00	100.00	PPV: 100.00/NPV: 96.77
	Observer 2	90.00	60.00	96.67	PPV: 80.00/NPV: 91.58
	Observer 3	87.27	60.00	93.33	PPV: 66.67/NPV: 91.30
Psoriasis	Observer 1	95.45	85.71	98.78	PPV: 96.0/NPV: 95.29
	Observer 2	78.18	64.29	82.93	PPV: 56.25/NPV: 87.18
	Observer 3	73.64	46.43	82.93	PPV: 48.15/NPV: 81.93
Eczema	Observer 1	92.73	98.39	85.42	PPV: 89.71/NPV: 97.62
	Observer 2	73.64	77.42	68.75	PPV: 76.19/NPV: 70.21
	Observer 3	62.73	69.35	54.17	PPV: 66.15/NPV: 57.78

### 3.3 Correlations of Disease‐Associated Histopathological Features

The features identified by observers were correlated with the corresponding findings in histopathology as a gold standard. The focus was on the main histological categories: stratum corneum, epidermis, inflammatory infiltrate and single cells. Observers assessed various features within these categories and their observations were compared to histopathological results using Cohen's kappa statistic to measure the level of agreement [21].

Focusing on single‐cell types (neutrophils, eosinophils, lymphocytes) all observers demonstrated almost perfect agreement with histopathology results (observer 1: *κ* = 0.89, 95% CI [0.81–0.96]; observer 2: *κ* = 0.85, 95% CI [0.76–0.93]; observer 3: *κ* = 0.80, 95% CI [0.71–0.90]).

When evaluating features of the epidermis (acanthosis, spongiosis, sawtooth‐like hyperplasia, psoriasiform hyperplasia, non‐specific hyperplasia or normal epidermis), observer 1 demonstrated the best performance, exhibiting substantial agreement with histopathological findings (*κ* = 0.73, 95% CI [0.64–0.83]). Observer 2 also showed substantial agreement (*κ* = 0.64, 95% CI [0.53–0.74]), while observer 3 attained moderate agreement (*κ* = 0.60, 95% CI [0.49–0.71]).

For inflammatory infiltrates (perivascular, band‐like, diffuse infiltrate or absence of infiltrate), observer 1 showed the highest level of agreement with histopathology (*κ* = 0.62, 95% CI [0.49–0.75]) compared to other observers. This was followed by observer 2 and observer 3, who demonstrated moderate agreement (observer 2: *κ* = 0.57, 95% CI [0.46–0.69]; observer 3: *κ* = 0.47, 95% CI [0.33–0.61]).

**FIGURE 2:**
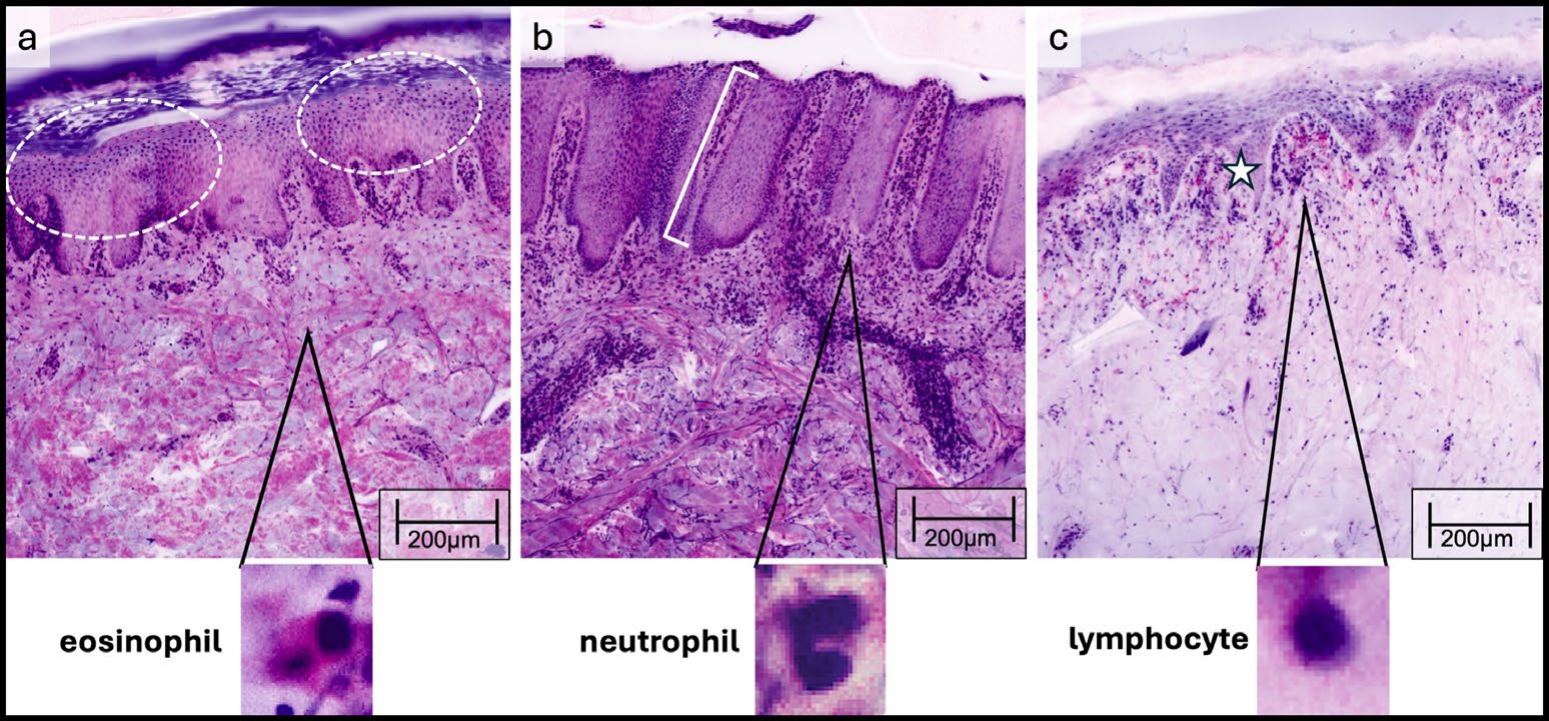
EVCM of inflammatory lesions in digital haematoxylin–eosin (DHE): Eczema with thickened epidermis, parakeratosis and spongiosis (white circles) (a); psoriasis (b) with elongated rete ridges and thinning of the suprapapillary plates (white bracket) (b); lichen planus with sawtoothing of rete ridges and band‐like lymphohistiocytic infiltrate obscuring the dermoepidermal junction (star) (c) as well as the prevalence of eosinophils and neutrophils in the magnification compared to gold standard H&E histopathology.

The features of the stratum corneum (hyperkeratosis, parakeratosis, orthokeratosis) were the least accurately recognized compared to histopathology. All observers attained fair agreement (observer 1: *κ* = 0.36, 95% CI [0.23–0.49], observer 2: *κ* = 0.37, 95% CI [0.22–0.52], observer 3: *κ* = 0.37, 95% CI [0.11–0.63], Figure 2).

### 3.4 Detailed Correlations of Disease‐Associated Histopathological Features

Table 2 presents the correlations between EVCM and histopathology, detailing the specific features identified within each main category of skin layer. Observer 1 demonstrated perfect agreement for the detection of sawtooth‐like hyperplasia (*κ* = 1.00, 95% CI [0.99–1.00]), almost perfect agreement for the observation of eosinophils (*κ* = 0.95, 95% CI [0.88–1.00]), psoriasiform hyperplasia (*κ* = 0.90, 95% CI [0.81–0.99]), neutrophils (*κ* = 0.88, 95% CI [0.78–0.97]) and lymphocytes (*κ* = 0.85, 95% CI [0.76–0.95]). Substantial agreement was also observed for band‐like infiltrate (*κ* = 0.71, 95% CI [0.53–0.89]), acanthosis (*κ* = 0.70, 95% CI [0.55–0.84]) and spongiosis (*κ* = 0.61, 95% CI [0.43–0.80]).

**TABLE 2:** Display of Cohen's kappa coefficients calculated to evaluate the agreement between cutaneous features detected in ex vivo confocal laser scanning microscopy (EVCM) images by observers and the gold standard histopathology, including the kappa values and their interpretations for each observer to indicate the level of agreement.

Feature		Cohen's Kappa	Interpretation
Parakeratosis	Observer 1	0.49 95% CI [0.32–0.65]	Moderate
	Observer 2	0.47 95% CI [0.29–0.65]	Moderate
	Observer 3	0.39 95% CI [0.19–0.60]	Fair
Hyperkeratosis	Observer 1	0.28 95% CI [0.10–0.45]	Fair
	Observer 2	0.26 95% CI [0.05–0.46]	Fair
	Observer 3	0.22 95% CI [0.01–0.42]	Fair
Orthokeratosis	Observer 1	0.27 95% CI [0.07–0.46]	Fair
	Observer 2	0.34 95% CI [0.12–0.56]	Fair
	Observer 3	—	—
Acanthosis	Observer 1	0.70 95% CI [0.55–0.84]	Substantial
	Observer 2	0.52 95% CI [0.34–0.70]	Moderate
	Observer 3	0.61 95% CI [0.45–0.78]	Substantial
Sawtooth‐like hyperplasia	Observer 1	1.00 95% CI [0.99–1.00]	Perfect agreement
	Observer 2	0.87 95% CI [0.73–1.00]	Almost perfect agreement
	Observer 3	0.86 95% CI [0.71–1.00]	Almost perfect agreement
Psoriasiform hyperplasia	Observer 1	0.90 95% CI [0.81–0.99]	Almost perfect agreement
	Observer 2	0.81 95% CI [0.69–0.92]	Almost perfect agreement
	Observer 3	0.76 95% CI [0.63–0.89]	Substantial
Spongiosis	Observer 1	0.61 95% CI [0.43–0.80]	Substantial
	Observer 2	0.63 95% CI [0.44–0.81]	Substantial
	Observer 3	0.45 95% CI [0.24–0.67]	Moderate
Band‐like inflammatory infiltrate	Observer 1	0.71 95% CI [0.53–0.89]	Substantial
	Observer 2	0.73 95% CI [0.56–0.91]	Substantial
	Observer 3	0.71 95% CI [0.53–0.89]	Substantial
Diffuse inflammatory infiltrate	Observer 1	0.59 95% CI [0.43–0.75]	Moderate
	Observer 2	0.56 95% CI [0.40–0.72]	Moderate
	Observer 3	0.38 95% CI [0.21–0.55]	Fair
Perivascular inflammatory infiltrate	Observer 1	0.55 95% CI [0.40–0.70]	Moderate
	Observer 2	0.67 95% CI [0.53–0.81]	Substantial
	Observer 3	0.38 95% CI [0.22–0.54]	Fair
Neutrophile granulocytes	Observer 1	0.88 95% CI [0.78–0.97]	Almost perfect agreement
	Observer 2	0.86 95% CI [0.76–0.96]	Almost perfect agreement
	Observer 3	0.79 95% CI [0.67–0.91]	Substantial
Eosinophile granulocytes	Observer 1	0.95 95% CI [0.88–1.00]	Almost perfect agreement
	Observer 2	0.87 95% CI [0.76–0.98]	Almost perfect agreement
	Observer 3	0.78 95% CI [0.64–0.92]	Substantial
Lymphocytes	Observer 1	0.85 95% CI [0.76–0.95]	Almost perfect agreement
	Observer 2	0.81 95% CI [0.71–0.92]	Almost perfect agreement
	Observer 3	0.84 95% CI [0.73–0.94]	Almost perfect agreement

Similarly, observer 2 achieved comparable results, exhibiting almost perfect agreement in correctly detecting sawtooth‐like hyperplasia (*κ* = 0.87, 95% CI [0.73–1.00]), eosinophils (*κ* = 0.87, 95% CI [0.76–0.98]), neutrophils (*κ* = 0.86, 95% CI [0.76–0.96]), lymphocytes (*κ* = 0.81, 95% CI [0.71–0.92]) and psoriasiform hyperplasia (*κ* = 0.81, 95% CI [0.69–0.92]). Substantial agreement was again evident for band‐like infiltrate (*κ* = 0.73, 95% CI [0.56–0.91]), perivascular infiltrate (*κ* = 0.67, 95% CI [0.53–0.81]) and spongiosis (*κ* = 0.63, 95% CI [0.44–0.81]).

Regarding observer 3, an almost perfect agreement was identified for sawtooth‐like hyperplasia (*κ* = 0.86, 95% CI [0.71–1.00]) and lymphocytes (*κ* = 0.84, 95% CI [0.73–0.94]). Additionally, substantial agreement could be noted for neutrophils (*κ* = 0.79, 95% CI [0.67–0.91]), eosinophils (*κ* = 0.78, 95% CI [0.64–0.92]), psoriasiform hyperplasia (*κ* = 0.76, 95% CI [0.63–0.89]), band‐like infiltrate (*κ* = 0.71, 95% CI [0.53–0.89]) and acanthosis (*κ* = 0.61, 95% CI [0.45–0.78]).

### 3.5 Logistic Regression Between Disease‐Associated Histopathological Features and Their Association With Correct Diagnoses

To investigate the relationship between histopathological features detected by the observers in EVCM images and their association with correct diagnoses, we employed Firth's multivariable logistic regression models. The independent variables in these models included characteristics of the stratum corneum, epidermis, inflammatory infiltrate and single‐cell types identified by the observers. The dependent variables were the correctly made diagnoses of eczema, psoriasis and lichen planus. By analysing how variations in the observed features (independent variables) influenced the likelihood of accurate diagnoses (dependent variables), we aimed to assess the impact of these histopathological characteristics on diagnostic accuracy. For each respective category, we designated the most frequently observed feature as the reference category. Specifically, parakeratosis was used as the reference for the stratum corneum, psoriasiform hyperplasia for the epidermis, perivascular infiltrate for the inflammatory infiltrate and lymphocytes for single‐cell types.

**FIGURE 3:**
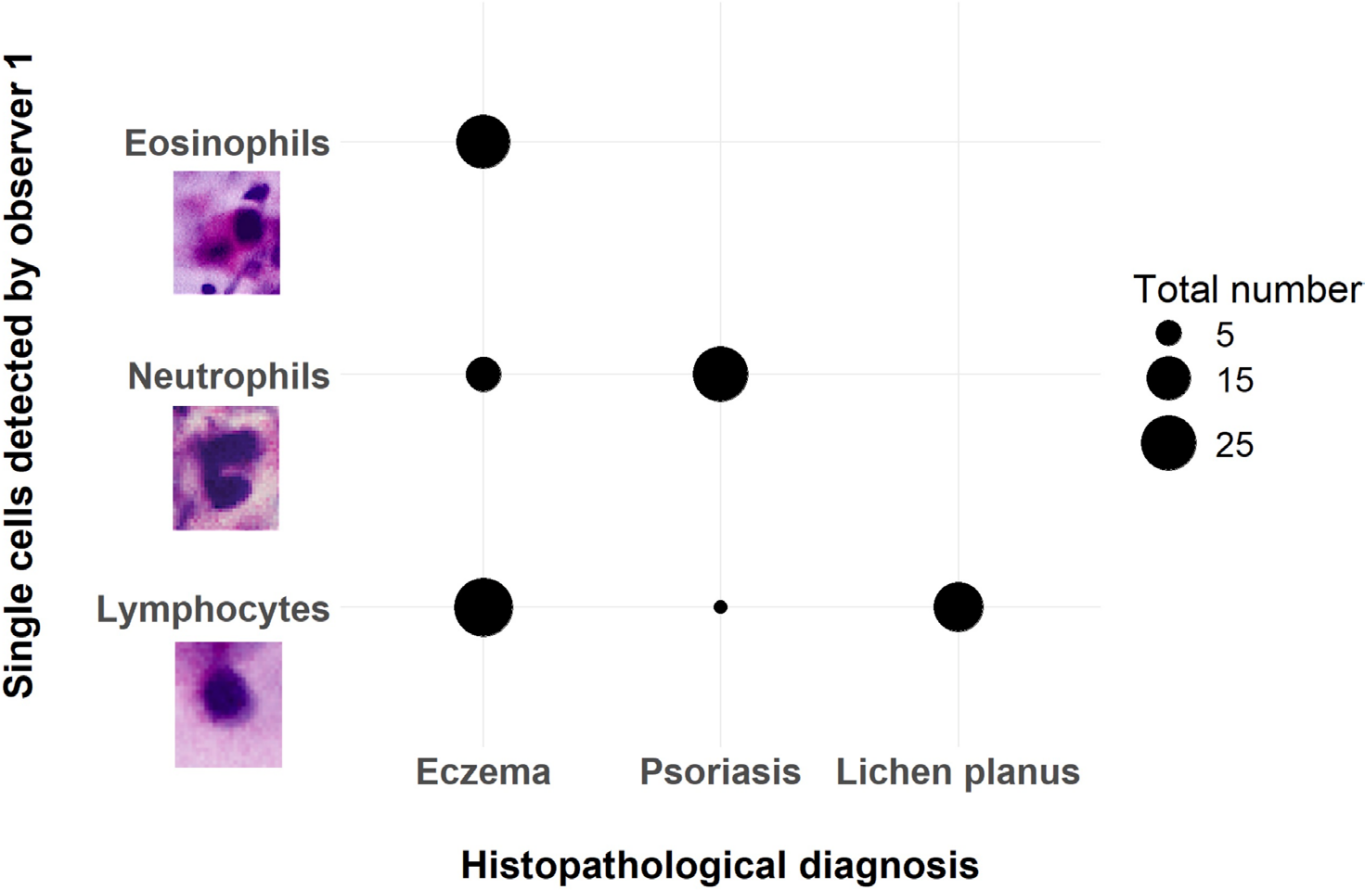
This plot shows the total number of single‐cell types detected by observer 1 in EVCM for each histopathological diagnosis. The bubble size indicates the total number of cells, allowing for easy comparison across the inflammatory diseases.

In the logistic regression model for observer 1, the detection of eosinophils (coef = 3.37, 95% CI [0.97–8.57], *p* < 0.01) and lymphocytes (coef = 2.55, 95% CI [1.21–4.20], *p* < 0.001) in EVCM was significantly associated with higher odds of accurately diagnosing eczema. Regarding psoriasis, observer 1 demonstrated significantly increased odds of a correct diagnosis when neutrophils were observed (coef = 4.02, 95% CI [1.94–8.95], *p* < 0.001, Figure 3). Additionally, when hyperplasia was identified as psoriasiform, the likelihood of correctly diagnosing psoriasis was higher compared to cases where hyperplasia was not assigned to a specific pattern (coef = 3.06, 95% CI [0.40–8.05], *p* = 0.02). Lastly, the observation of a band‐like inflammatory infiltrate was significantly linked to higher odds of accurately diagnosing lichen planus (coef = 2.53, 95% CI [0.50–4.90], *p* = 0.02). The regression models for observer 2 and 3 showed similar results (Table S2).

## 4 Discussion

The findings of this study highlight the potential of EVCM as a valuable diagnostic tool for inflammatory dermatological diseases, specifically eczema, psoriasis and lichen planus. The utilisation of both pattern analysis according to Ackermann's classification and single‐cell analysis of neutrophils, eosinophils and lymphocytes contributes to a comprehensive understanding of the diagnostic capabilities of EVCM.

To our knowledge, four publications have addressed the use of EVCM in diagnosing inflammatory skin diseases [23, 24, 25, 26]. We expand upon this prior research by including a larger sample size and emphasising single‐cell analysis, offering a more detailed exploration of cellular patterns and their diagnostic relevance.

To simulate a real‐world clinical setting, no images were excluded, and all acquired EVCM images were analysed even with poor image quality. Fixation or staining artefacts may account for the moderate to fair agreement observed in the correlation with histopathology, particularly regarding the visualisation of the stratum corneum and inflammatory infiltrate. Nevertheless, the distinctive patterns observed in EVCM images align with established histopathological features, underscoring the ability of EVCM to provide rapid and meaningful visualisation of characteristic disease features in a real‐life setting. Furthermore, the rapid acquisition of images allows for on‐the‐spot analysis, potentially expediting the diagnostic process compared to traditional histopathology.

Ackermann's classification, applied to EVCM pattern analysis, aided in identifying disease‐associated features. This classification provided a practical framework for differentiating eczema, psoriasis and lichen planus based on specific architectural and cellular alterations observed in the skin. Moreover, the quantification and characterisation of neutrophils and eosinophils through single‐cell analysis added granularity to the evaluation of inflammatory infiltrates. This level of detail can contribute to a more nuanced understanding of the immune response in different dermatological conditions. The comparison of EVCM results with histopathology as the gold standard demonstrated promising diagnostic accuracy. Sensitivity and specificity values indicated the potential for EVCM to reliably identify and differentiate inflammatory skin diseases, laying the groundwork for its integration into routine clinical practice.

Our results suggest that the diagnostic accuracy in EVCM is influenced by the observer's experience in both EVCM and histopathology. The highest diagnostic accuracy was achieved by the observer proficient in both disciplines, followed by observer 2, who is experienced in EVCM only.

Firth's multivariable logistic regression models revealed that the detection of specific features in EVCM images was significantly associated with higher diagnostic accuracy. Specifically, the observation of eosinophils and lymphocytes increased the odds of correctly diagnosing eczema, while the identification of neutrophils and psoriasiform hyperplasia was strongly linked to accurate psoriasis diagnoses. Furthermore, recognising a band‐like inflammatory infiltrate enhanced the likelihood of correctly identifying lichen planus. The identified features are well‐established indicators of the three diseases in histopathology. These results suggest that EVCM can reliably detect these features, which may support more accurate diagnoses.

The promising results of this study lay the foundation for future development of AI‐based algorithms to assist in the automated diagnosis of inflammatory skin diseases. Such algorithms could help observers identify specific patterns and cell types with higher accuracy, ultimately leading to more reliable diagnoses. Future advancements in this field could be implemented in upcoming studies, further enhancing diagnostic precision and efficiency.

While acknowledging the study's limitations, particularly its focus on specific inflammatory dermatological diseases, the findings lay a foundation for future research. Broader investigations involving a wider range of dermatopathological conditions and larger patient cohorts are warranted. Future studies should also explore the feasibility of integrating EVCM into routine clinical practice to assess its utility and performance in diverse clinical settings.

## 5 Conclusion

In conclusion, this study highlights the potential of EVCM as a rapid and insightful imaging technique for inflammatory dermatological diseases. The combination of pattern analysis and single‐cell analysis enhances the diagnostic value of EVCM, positioning it as a promising adjunct to traditional histopathology to improve patient care in the realm of inflammatory dermatological diseases. Continued research and validation efforts are essential to reinforce the role of EVCM in routine dermatological practice.

## Author Contributions

Conceptualization: D.H. and M.D.; methodology: D.H., L.S. and M.D.; investigation: D.H., L.S., L.B., A.S. and M.D.; data curation: D.H., L.S., M.M. and M.D.; formal analysis: D.H., L.S., Y.S. and M.D.; visualisation: L.S., Y.S. and M.D.; writing – original draft: L.S. and M.D.; writing – review and editing: D.H., M.M., Y.S., L.B, A.S., E.C.S. and L.E.F.; supervision: D.H., E.C.S., L.E.F. and M.D.

## Ethics Statement

The study was approved by the local ethics committee of the LMU University Hospital (Ref.‐Nr. 19‐150 and Ref.‐Nr. 23‐0393).

## Consent

Informed consent was obtained from all patients involved in the study.

## Conflicts of Interest

The authors declare no conflicts of interest.

## Supporting information


**Table S1:** Descriptive statistics of all patients, including age, sex and biopsy localization, categorised by psoriasis, eczema and lichen planus. Normal distributed metric variables are reported as mean ± standard deviation and non‐normal distributed metric variables as median (interquartile range).
**Table S2:** This table presents regression models of EVCM features and their association with accurate histopathological diagnosis across the three observers. Predictors include specific features of the stratum corneum, epidermis, inflammatory patterns and single cells. The coefficient represents the logarithm of the Odds Ratio (log(OR)), as well as the 95% confidence interval and *p*‐value.

## Data Availability

The data that support the findings of this study are available from the corresponding author upon reasonable request.
