# Supplementary material for: Ex Vivo Confocal Laser Scanning Microscopy for Real‐Time Pattern and Single‐Cell Analysis in Inflammatory Skin Diseases
Source: Exp Dermatol. 2025 Nov 17;34(11):e70179. doi: 10.1111/exd.70179 (PMC12623711; doi:10.1111/exd.70179)
Supplement: Supplementary file 1 — Table S1: Descriptive statistics of all patients, including age, sex and biopsy localization, categorised by psoriasis, eczema and lichen planus. Normal distributed metric variables are reported as mean ± standard deviation and non‐normal distributed metric variables as median (interquartile range). Table S2: This table presents regression models of EVCM features and their association with accurate histopathological diagnosis across the three observers. Predictors include specific features of the stratum corneum, epidermis, inflammatory patterns and single cells. The coefficient represents the logarithm of the Odds Ratio (log(OR)), as well as the 95% confidence interval and p‐value. [file EXD-34-e70179-s001.docx]

## Supplementary material

**Supplementary Table 1** - Descriptive statistics of all patients, including age, sex and biopsy localization, categorized by psoriasis, eczema and lichen planus. Normal distributed metric variables are reported as mean ± standard deviation and non-normal distributed metric variables as median (interquartile range).

| Age at biopsy (years) | | | | | |
| --- | --- | --- | --- | --- | --- |
|  |  | 53.84 (36.95 – 63.92) | | | |
| Sex (n) |  |  |  |  |  |
|  | Female | 58 |  |  |  |
|  | Male | 52 |  |  |  |
| Localization of biopsy (n) | | | | | |
|  | Head | 9 |  |  |  |
|  | Thorax | 37 |  |  |  |
|  | Arm | 32 |  |  |  |
|  | Leg | 32 |  |  |  |
|  |  | **Psoriasis** | **Eczema** | **Lichen planus** | **p-value** |
| Age |  | 51.28 ± 17.1 | 51.30 ± 18.55 | 49.21 ± 18.83 | 0.85 ^a^ |
| Diagnosis observer 1 (n) | | 25 | 68 | 17 | - |
| Diagnosis observer 2 (n) | | 32 | 63 | 15 | - |
| Diagnosis observer 3 (n) | | 27 | 65 | 18 | - |
| Diagnosis histopathology (n) | | 28 | 62 | 20 | - |
| Sex (n) |  |  |  |  |  |
|  | Female | 15 | 33 | 10 | 0.96 ^b^ |
|  | Male | 13 | 29 | 10 |  |
| Localization of biopsy (n) | |  |  |  |  |
|  | Head | 0 | 7 | 2 | 0.19 ^c^ |
|  | Thorax | 12 | 17 | 8 |  |
|  | Arm | 5 | 22 | 5 |  |
|  | Leg | 11 | 16 | 5 |  |

**^a^** *Kruskal-Wallis rank sum test*

**^b^** *Chi-Square test (Χ²)*

**^c^** *Fisher's Exact Test*

**Supplementary Table 2 -** This table presents regression models of EVCM features and their association with accurate histopathological diagnosis across the three observers. Predictors include specific features of the stratum corneum, epidermis, inflammatory patterns and single cells. The coefficient represents the logarithm of the Odds Ratio (log(OR)), as well as the 95% confidence interval and p-value.

| Observer 1 | | | | |
| --- | --- | --- | --- | --- |
| EVCM-observed feature | **Correctly diagnosed disease** | **Coefficient** | **95 % CI** | **p-value** |
| Neutrophils | Psoriasis | 4.02 | [1.94 – 8.95] | < 0.001 |
| Psoriasiform hyperplasia/ non-specific hyperplasia | Psoriasis | 3.06 | [0.40 – 8.05] | 0.02 |
| Eosinophils | Eczema | 3.37 | [0.97 - 8.57] | < 0.01 |
| Lymphocytes/ neutrophils | Eczema | 2.55 | [1.21 – 4.20] | < 0.001 |
| Band-like inflammatory infiltrate | Lichen planus | 2.53 | [0.50 - 4.90] | 0.02 |
| Observer 2 | | | | |
| EVCM-observed feature | **Correctly diagnosed disease** | **Coefficient** | **95 % CI** | **p-value** |
| Neutrophils | Psoriasis | 3.39 | [0.90 - 8.58] | < 0.01 |
| Lymphocytes/ neutrophils | Eczema | 1.76 | [0.01 - 3.71] | 0.04 |
| Observer 3 | | | | |
| EVCM-observed feature | **Correctly diagnosed disease** | **Coefficient** | **95 % CI** | **p-value** |
| Neutrophils | Psoriasis | 4.99 | [1.27 - 14.52] | < 0.01 |
| Acanthosis | Eczema | 2.88 | [0.97 - 5.96] | < 0.01 |
| Perivascular inflammatory infiltrate / band-like infiltrate | Eczema | 2.62 | [0.28 – 6.11] | 0.03 |
| Eosinophils | Eczema | 2.33 | [0.59 - 5.11] | < 0.01 |
| Lymphocytes/ neutrophils | Eczema | 2.81 | [0.27 – 8.92] | 0.03 |
